# Supplementary material for: Experience and satisfaction towards palliative care in an Ethiopian tertiary care setting: A mixed methods study of patients with cancer and caregivers
Source: PLOS Glob Public Health. 2026 Apr 21;6(4):e0005754. doi: 10.1371/journal.pgph.0005754 (PMC13098945; doi:10.1371/journal.pgph.0005754)
Supplement: S1 Appendix — (DOCX) [file pgph.0005754.s001.docx]

1. **Qualitative Data collection tools**

**Section I: Information sheet and consent form**

**Information sheet**

**Introduction**: Palliative care as defined by the World Health Organization (WHO) is “an approach that improves the quality of life of patients and their families facing the problem associated with life-threatening illness through prevention and relief of suffering by means of early identification and impeccable assessment and treatment of pain and other problems-physical, psychosocial, and spiritual. Each year, an estimated 56.8 million people, including 25.7 million in the last year of life, are in need of palliative care. However, only about 14% of people who need palliative care currently receive it globally. Access to morphine and other palliative care medications and week health system structure are the major hindering factors of palliative care. Hence, this PhD research project entitled “**Design, Implementation, and Evaluation of a Multidisciplinary Palliative Care Service among patients treated for cancer in Tikur Anbessa Specialized Hospital, Ethiopia**” designed to understand and address the palliative care problem in TASH and Ethiopia at large. This form will help you decide whether or not to participate in this research study. The person performing the research will answer any of your questions. Read the information below and ask any questions you might have before deciding to take part in the study or not. If you decide to be involved in this study, this form will be used to record your consent.

**Purpose of the Study:** You have been asked to participate in a research study about palliative care service in Ethiopia and at Tikur Anbessa Specialized Hospital in particular. The purpose of this study is to assess and understand the overall burden of palliative care service, barriers and facilitators of palliative care, and then develop palliative care guideline, training manuals, implement multidisciplinary palliative care service at TASH and assess its impact on clinical, humanistic and economic outcomes, etc. The findings will have impact on cancer care and policy implication in Ethiopia to improve palliative care service throughout the country. The ultimate objective of the study is to improve palliative care in TASH and expand the service to other cancer care centers/hospitals throughout the country.

**Procedure:** In order to collect our data, we invite you to take part in the study. If you are willing to participate, you need to understand the purpose of the study and sign on the consent form. If you agree to participate in the study, we will ask you to answer some questions about palliative care and the current service challenges and facilitators. Extracts from this study may be quoted in the study report, presentations and any subsequent publications.

**Risk and /or discomfort:** There is minimal risk in participating in this study. However, you will spend about 30 minutes for helping us respond to our questions.

**Benefits:** Potential benefits of this study is primarily to patients that would be receiving palliative care and the society through improved palliative care. This study will benefit the society at-large and patients in particular by providing inputs for improving relevant evidence for healthcare providers, hospital administrators, policymakers, and researchers on chemotherapy market, quality and palliative care.

***Incentives/payments for participating***: We will not pay you for participating in the study; your participation is based on full understanding of the purpose of the study and your willingness.

**Confidentiality:** The information collected from this research project will be kept confidential and information about you that will be collected by this study will be stored in a file, without your name and will be accessed only by the research team members.

**Right to refusal or withdraw:** Your participation is purely based on your willingness. You have the full right to either participate or to decline your participation in this study. If you choose to take part in the study, you may respond to all the questions or you may not answer questions you don’t want to, and have the right to stop at any time. You also have the right to choose not to take part in this study. However, because the information you will be providing us is very useful for the study, we encourage you to participate and provide us with the information we need.

If you want to know more about the study, you can contact the investigator: Atalay Mulu Fentie (PhD student), e-mail: [**atalay.mulu@aau.edu.et**](mailto:atalay.mulu@aau.edu.et) , Tel. +251923295462; OR Prof. Teferi Gedif (PhD, Professor of Social and Administrative Pharmacy), Tel. +251911684854, email: [tgedif@gmail.com](mailto:tgedif@gmail.com) IRB of CHS, AAU by Tel: 251-115538734, e-mail: [aaumfirb@yahoo.com](mailto:aaumfirb@yahoo.com)

Do you have any questions?

**Consent form**

**Title:**  Design, implementation and evaluation of multi-disciplinary team palliative care for patients treated with cancer at Tikur Anbessa Specialized Hospital, Ethiopia.

**Principal Investigator and contacts:** Atalay Mulu Fentie (PhD candidate), e-mail: [**atalay.mulu@aau.edu.et**](mailto:atalay.mulu@aau.edu.et) , Tel. +251923295462; School of Pharmacy, CHS, Addis Ababa University, Addis Ababa, Ethiopia.

**Data collector:** Please provide a paper copy of the Information Sheet to the respondent and adequately explain it to her/him.

**Respondent:** I agree to participate in this study with my signature hereunder I confirm that:

- I am at least 18 years of age.
- The purpose and nature of the study has been explained to me in writing.
- I am participating voluntarily.
- I understand that I can withdraw from the study, without repercussions, at any time, whether before it starts or while I am participating.
- I understand that I can withdraw permission to use the data within two weeks of the interview, in which case the material will be deleted.
- I understand that anonymity will be ensured in the write-up to disguise my identity.
- I understand that disguised extracts from my study participation may be quoted in the study report, presentations and any subsequent publications if I give permission below: If yes, proceed. If No, End here
- If the answer is yes, do you want to be audio-recorded for the study? Yes / No ; If No, get permission to take notes.

_________________________________

Printed Research Participant’s Name

_________________________________ _________________

Signature Date

By signing this form, you are not waiving any of your legal rights.

As a representative of this study, I have explained the purpose, procedures, benefits, and the risks involved in this research study._________________________________

Print Name of Person obtaining consent

_________________________________ _________________

Signature of Person obtaining consent Date

- 1. **Interviewer guide (Patients/care-givers)**

**Participants’ background information**

| Respondent ID **__________** | Sex**:**  Male  Female |
| --- | --- |
| **Age in years**_______________ | **Address:**  Addis Ababa  Out of Addis Ababa |
| Highest educational level completed ____________ | Source of payment for the care (out of pocket, insurance, employer)_______ |
| Cancer diagnosis (site)___________ | Duration since cancer care follow-up started in months________________ |
| Type of treatment (Chemo/RT/Surgery/combination, not yet started) ______________ | |

**If you would like to receive information on the study kindly provide the following details.**

Participant email address: _______________________ Phone number: ___________________

**Interview guide**

1. Overall, how do you describe the service you are getting?
2. How do you describe the palliative care service you are getting in TASH?
   1. Satisfaction
   2. Availability of dedicated care
   3. Quality of the care
   4. Communication/professionals attention?
3. Can you tell me your palliative care concerns you have experienced?
   - - Medication availability?
     - The care and its quality you are receiving?
4. What problems/challenges have you faced related to your palliative care?
   1. How did you manage it?
5. How did you describe your satisfaction towards the palliative care you are receiving?
6. Your recommendations to improve the palliative care you are currently receiving?
7. From your point of view, are there any issues that you feel should be addressed, but weren’t mentioned thus far?

**Thank you very much for your time and consideration**
