# Supplementary material for: Experience and satisfaction towards palliative care in an Ethiopian tertiary care setting: A mixed methods study of patients with cancer and caregivers
Source: PLOS Glob Public Health. 2026 Apr 21;6(4):e0005754. doi: 10.1371/journal.pgph.0005754 (PMC13098945; doi:10.1371/journal.pgph.0005754)

**S2 Appendix- reliability and validity tests**

**Table A: Reliability of SATMED-Q**

| **Domain (6-subdomains)** | Number of items | Cronbach-α (stand.) | Item | Item correlation | Cronbach-α if item deleted |
| --- | --- | --- | --- | --- | --- |
| Undesirable side effects on treatment satisfaction (SAT_SEs) | 3 | 0.914 | SEs interfere on physical activity | 0.696 | 0.860 |
|  |  |  | SEs interference on leisure/free time activities | 0.876 | 0.852 |
|  |  |  | SEs on daily activities | 0.856 | 0.852 |
| Satisfaction on treatment effectiveness (SAT_ Effectiveness) | 3 | 0.971 | My treatments reduced symptoms | 0.814 | 0.817 |
|  |  |  | Satisfied for the treatment started to have an effect | 0.879 | 0.816 |
|  |  |  | Feel better now than previous | 0.876 | 0.814 |
| Satisfaction on convenience or ease of treatments (SAT_ Convenience) | 3 | 0.957 | Treatment is convenient to take | 0.839 | 0.832 |
|  |  |  | Treatment is easy to use | 0.826 | 0.830 |
|  |  |  | Timetable for the treatment is suitable | 0.673 | 0.828 |
| Satisfaction on treatment impact on daily livings (SAT_Impact) | 3 | 0.936 | I can undertake my leisure and free time activities. | 0.844 | 0.817 |
|  |  |  | I can more easily look after my personal hygiene | 0.732 | 0.820 |
|  |  |  | I can perform my everyday chores better. | 0.875 | 0.816 |
| Opinion on continuing medical care follow-up (SAT_care follow-up) | 2 | 0.985 | Received detailed information about my medical condition | 0.946 | 0.833 |
|  |  |  | Received right information to treat my medical condition | 0.947 | 0.833 |
| Global satisfaction (SAT_Global) | 3 | 0.829 | Will continue the same treatment | 0.348 | 0.831 |
|  |  |  | Feel happy with the treatment | 0.903 | 0.828 |
|  |  |  | Feel satisfied with treatment | 0.908 | 0.826 |
| Overall/composite satisfaction | **17** | **0.840** |  |  |  |

**Table B: Known group validity**

| **Overall satisfaction domain** | Pain severity, Mean ± SD | | | p-value |
| --- | --- | --- | --- | --- |
|  | Mild | Moderate | Severe |  |
| SAT_SEs | 65.29 ±28.7 | 52.94 ±26.32 | 49.54 ± 25.9 | <0.0001 |
| SAT_ Effectiveness | 61.15±32.0 | 47.37 ±31.9 | 44.46±32.16 | <0.0001 |
| SAT_ Convenience | 65.89 ±31.8 | 60.44 ±28.97 | 47.76±30.0 | <0.0001 |
| SAT_care follow-up | 74.84 ± 29.4 | 64.86 ± 30.7 | 57.29±37.2 | <0.0001 |
| SAT_Global | 76.44 ±19.8 | 69.89 ±20.1 | 62.52±23.8 | <0.0001 |
| Overall Satisfaction | 61.12 ± 18.1 | 52.64 ± 18.3 | 48.47±19.3 | <0.0001 |

**Appendix A: Measures of fit index and Construct validity**

| Measures of fit indices of latent constructs | fit indices |
| --- | --- |
| RMSEA | 0.069 |
| SRMR | 0.066 |
| TLI | 0.930 |
| RFI | 0.916 |
| CFI | 0.946 |
| IFI | 0.946 |

**
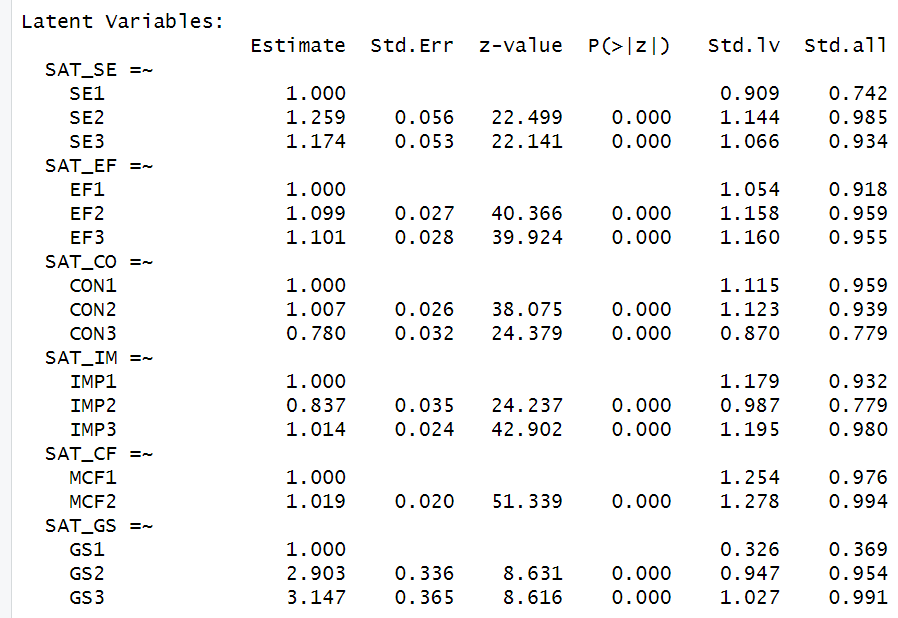
**

**
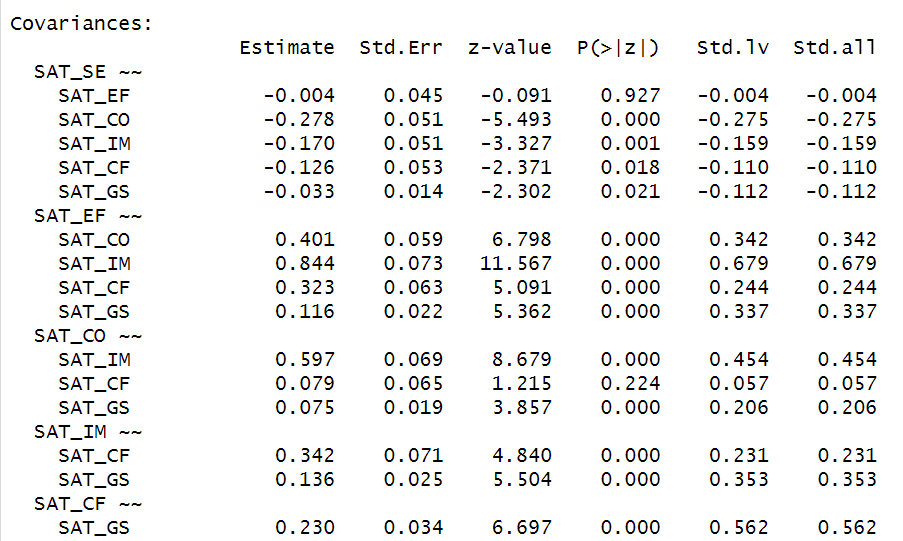
**

**
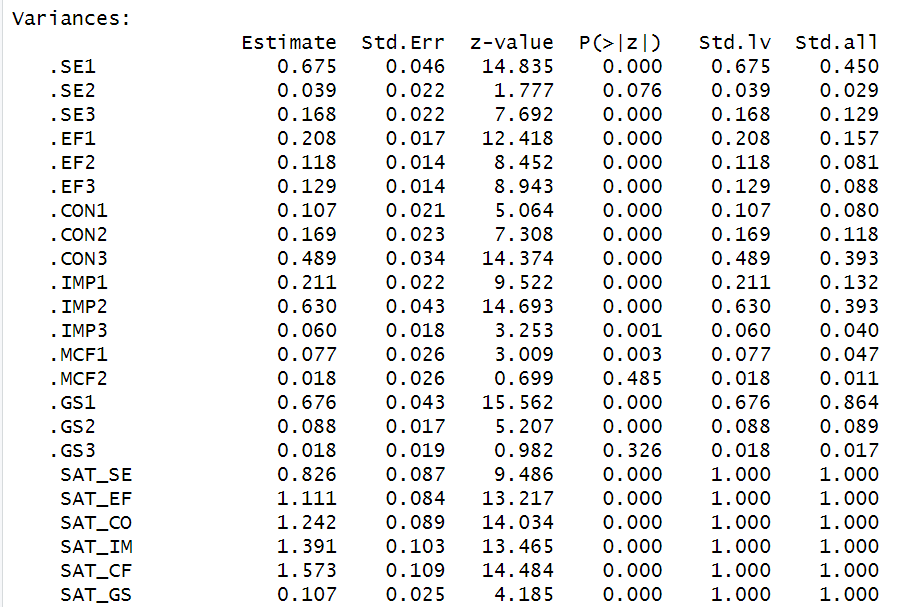
**


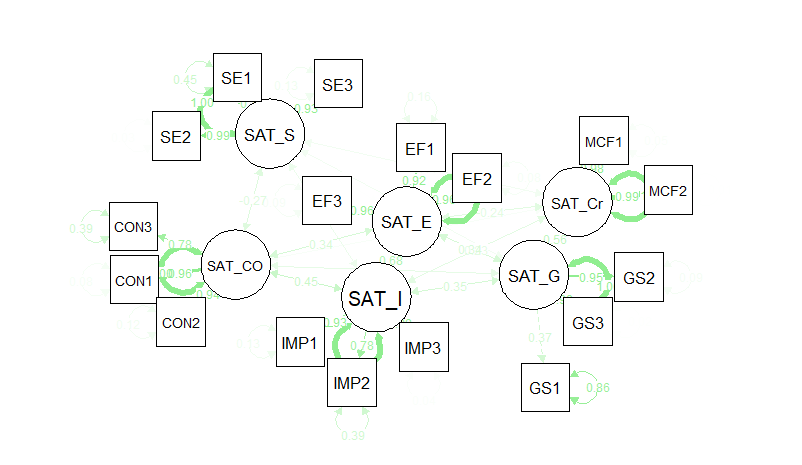

Supplement: S2 Appendix — (DOCX) [file pgph.0005754.s002.docx]
