# Supplementary material for: Experience and satisfaction towards palliative care in an Ethiopian tertiary care setting: A mixed methods study of patients with cancer and caregivers
Source: PLOS Glob Public Health. 2026 Apr 21;6(4):e0005754. doi: 10.1371/journal.pgph.0005754 (PMC13098945; doi:10.1371/journal.pgph.0005754)
Supplement: Attachment — Submitted filename: Point by point response letter PLOS Global Health.docx [file pgph.0005754.s005.docx]

**Date: December 16, 2025**

**Point by point response**

**To	PLOS Global Public Health**

**Dear Esteemed Editor and Reviewers,**

We would like to express our sincere gratitude for your thoughtful and constructive comments on our manuscript entitled, ***“Experience and satisfaction towards palliative care in an Ethiopian tertiary care setting: A mixed methods study of patients with cancer and caregivers”.***

We have carefully reviewed and addressed each of the comments provided, and we believe your feedback has greatly improved the quality and clarity of our manuscript. In response, we have revised the manuscript accordingly and prepared a point-by-point summary of our responses for your review.

To facilitate your evaluation, we attached the revised manuscript using track changes, file labeled 'Revised Manuscript with Track Changes' uploaded in related file section and a separate an unmarked version file labeled 'Manuscript', so you can easily identify the modifications made throughout the document.

We appreciate your time and effort in reviewing our work and look forward to your further guidance and feedback.

With kind regards,

| **Comment** | **Response** |
| --- | --- |
| **Editor comments** |  |
| 1. Please send a completed 'Competing Interests' statement, including any COIs declared by your co-authors. If you have no competing interests to declare, please state "The authors have declared that no competing interests exist". Otherwise please declare all competing interests beginning with the statement "I have read the journal's policy and the authors of this manuscript have the following competing interests:" | Thank you for your valuable feedback and the opportunity to revise our manuscript. Since all authors have declared that they have no competing interests, we revised the section to read **“**The authors have declared that no competing interests exist". |
| 2. Please amend your detailed Financial Disclosure statement. This is published with the article. It must therefore be completed in full sentences and contain the exact wording you wish to be published. | Thank you so much once again and we revised the financial disclosure section as follows. “This study was supported by the Pfizer Global Medical Grants (Grant #76384195) and by the Addis Ababa University Thematic Research Grant (Grant #RD/LT-305/2022). The funders had no role in study design, data collection and analysis, decision to publish, or preparation of the manuscript. AMF and ES received salary from support from Pfizer Global Medical Grants (Grant #76384195). |
| 3. Please provide separate figure files in .tif or .eps format. | We already provided the figure separately in tif format. |
| 4. Though the paper is well written, this being a mixed-methods research, integration of the quantitative and qualitative results should be done. | Thank you for your thoughtful observation. We agree that triangulation is an essential component of mixed-methods research and now presented the data triangulation in **Table 6 (Line number 399-407).** |
| **Reviewer #1** |  |
| 1. Introduction: Line 88 has typographical error. | Thank you for your valuable feedback and the opportunity to revise our manuscript. Now changed “the Ethiopia context” to “the Ethiopian context” (line number 100 of the clean version manuscript) |
| 2. Line 117: Sample size calculation of quantitative part was unclear. | Thank you for this helpful comment. We agree that the sample size calculation required further clarification. We have now revised the manuscript to provide a more detailed and scientifically sound description of the approach used; and revised as follows “Since no similar study had been conducted in the country to provide an estimate of the satisfaction level, we used a proportion of 50% to obtain the maximum possible sample size. The sample size was calculated using the single population proportion formula (20), with a 95% confidence level, 0.05 margin of error, and giving an initial estimate of 384 participants**.** Accounting for 10% non-responses rate and inappropriate responses as well as a design effect of 2 to account for the multistage sampling approach (initially at department level and then at outpatient and inpatient level), the study finally recruited a total of 844 patients. The revised text now appears in **Line number** **143-149 of the manuscript.** |
| 3. Table 1: Add N for Type of comorbidity | Thank you so much and now included the number in the comorbidity row. |
| 4. Patients residing outside of Addis Ababa is general statement. The characteristics of such classification like how much distance away from TASH, absence of tertiary hospitals in surrounding radius would make it clear to ascertain the satisfaction and to avoid confounders. | Thank you for this valuable comment. We agree that distance from the hospital is an important factor and that further clarification was needed. During the study design phase, we initially considered using the actual distance from Addis Ababa or from TASH to categorize participants. However, during data collection, we found that a substantial proportion of participants were unable to accurately estimate the distance from their residence to the hospital, making the measure highly unreliable and prone to misclassification. Although distance is indeed an important determinant of satisfaction; particularly due to travel time, transportation availability, and associated costs; the research team determined that a more reliable and feasible approach was to dichotomize residence as “within Addis Ababa” versus “outside Addis Ababa.” This classification is meaningful for several reasons:   1. **TASH is located in Addis Ababa**, and patients residing within the city have substantially different access patterns, transportation costs, and service availability compared with those coming from outside the city. 2. **Previous studies conducted in Ethiopia** have demonstrated that residence outside Addis Ababa, *regardless of the exact distance*, is an independent predictor of patient satisfaction, particularly due to structural barriers, referral complexities, and socioeconomic factors. 3. Using a dichotomous classification reduced the risk of recall bias and misclassification, while still capturing the key contextual difference that influences satisfaction in this setting. |
| 5. The details on palliative care services in the setting which was provided to the patients was not explained. Whether the patients who were given curative treatment provided with palliative care could be explained to understand the situation. | Thank you for this important comment. We agree that clearer description of the palliative care services available in the study setting is essential for proper interpretation of the findings. We have revised the Methods section and included the same under the study **setting from line number109-124.** |
| 6. Line 262-269: The authors explanation of association with various cancers and pain is appreciated taking into consideration the scientific way of explain the multiple linear regression output but it looks more statistical and can be modified. “Every additional patient” doesn’t make sense in this study point and can be explained better. | Thank you so much for the constructive comment. We agree that the original wording was overly statistical and that the phrase “every additional patient” was inappropriate. We have revised the text to provide a clearer, clinically meaningful interpretation as follows: “Keeping other predictors constant, patients who came from outside Addis Ababa a significantly lower satisfaction (β = –2.77, 95% CI: –5.29, –0.25; *p* = 0.03) as compared to patients living in Addis Ababa where the treatment center is located. Similarly, patients with cancer of the reproductive system (β = -6.64, 95%CI: -10.38, -2.91, *p<*0001), GIT (β = -4.11, 95%CI:-7.85, -0.38, *p=*0.031), head and neck (β = -11.56, 95%CI: -16.63, -6.50, *p*<0.001), lung or mediastinum (β = -9.96, 95%CI: -16.63, -6.50, *p*<0.001), genitourinary system (β = -7.95, 95%CI: -14.50, -1.40, *p*=0.017), musculoskeletal system (β = -12.15, 95%CI:-18.02, -6.27, *p*<0.001) and central nervous system (β = -17.83, 95%CI: -32.54, -3.14, *p=*0.018) reported a substantially lower satisfaction scores when compared with patients with breast cancer.  Additionally, patients experiencing moderate or severe pain had markedly lower satisfaction levels, with mean scores reduced by 7.34 percentage points (*p*< 0.001) and 11.5 percentage points (*p*< 0.001), respectively, compared to those with mild or no pain.” **(Line number 291-302)** |
| 7. The satisfaction scores in unemployed were better compared with employed and that could have been explored in qualitative. The qualitative part is written well but it needs some refinement to explain the quantitative results. | Thank you for this insightful comment. Although the analysis showed slightly higher satisfaction scores among unemployed participants, this difference was not statistically significant. Now, we contextually explained in the data triangulation section to more explicitly contextualize the overall quantitative findings to highlight areas where qualitative data meaningfully explain statistically supported results (**Table 6, line number 399-407)**. |
| 8. The Amharic text font is not supported. Try to correct it and make it visible. | Thank you so much once again and now we changed the font type. |
| 9. The biases involved in selection of participants could be mentioned. | Thank you for the comment. We have acknowledged the potential for **selection bias** in participant recruitment in the Limitations section, noting that purposive sampling may have influenced the representativeness of the study population and, consequently, the generalizability of the findings. Now included the same in the discussion section, last **paragraph from line 498-501.** |
| Reviewer #2 |  |
| 1. Details of the palliative care services which are currently being provided are missing. Another crucial detail is how many are aware of these services, how many are eligible for these services, and how many availed these services? Then the satisfaction will be measured among those who all in need tried to utilise the services. | Thank you very much for your valuable and constructive comments, and opportunity to revise the manuscript. In response to Reviewer 1’s suggestion, we have now incorporated the requested information into the **Methods section**, specifically under the **“Study Setting”** subsection, to provide a clearer description of the context and services (**line number 109-124)** |
| 2. Details about how many patients approached, how many refused to participate. There would patients who could not comprehend the questionnaire due to the severity of illness or distress, as 10.9% of patients had the status of ‘couldn’t function’. | Thank you for this important comment. A total of **879 eligible patients** (both inpatients and outpatients) were approached for the study, of whom **844 completed the survey**, resulting in a **response rate of 96.1%**. Refusal to participate was more common among outpatients, primarily due to overcrowding and long waiting times. Recruitment for the qualitative component was more challenging, as some patients and caregivers declined participation for various reasons, including concerns that their care might be affected, burnout, or other personal constraints, often without allowing time for study explanations or reassurances. We have added this information to the **Results section (line number 239-240)** for clarity: *“About 879 eligible patients were approached for the study, and a total of 264 inpatients and 580 outpatients completed the survey, giving a response rate of 96.1%.”* |
| 3. Satisfaction measured among patients was measured objectively, while the satisfaction of caregivers is missing, as both have a very different journey and expectations in the hospital, which affects the majority’s satisfaction. | Thank you for this insightful comment. We acknowledge that caregiver satisfaction was not measured using a structured or quantitative tool, unlike patient satisfaction, which may limit direct comparison between the two groups. However, caregivers’ perspectives were captured qualitatively through interviews, providing rich contextual insights into their experiences and expectations, which complement the patient satisfaction data. We have now included this as a limitation in the last paragraph of the Discussion, as follows: *“Caregiver satisfaction was not measured quantitatively, and future studies should consider structured assessment of both patients’ and caregivers’ satisfaction to better capture differences in experiences and expectations.”****(line number 504-506).*** |
| 4. While qualitative research methodology is used, triangulation is missing. Here, utilizing a focus group discussion instead of in-depth interviews would have given a better generation of data. | Thank you for this important comment. We have now explicitly addressed data triangulation by including a separate **“Data Triangulation”** subsection in the Results. In this subsection, we summarize the convergence and complementarity of qualitative and quantitative findings, with key points presented in **Table 6** for clarity (**line number 399-407)**. While we acknowledge that FGDs could have provided additional group-level insights, conducting FGDs in the hospital setting was not feasible due to logistical constraints, scheduling difficulties, and the clinical condition of participants. In-depth interviews were therefore chosen to ensure participants could freely share personal and sensitive experiences, while still providing rich qualitative data to complement the quantitative findings. |
| 5. Adding the perspective of healthcare providers and administration would have given a 360-degree picture of the situation. As patient satisfaction is just a tip of the iceberg. | Thank you for this valuable comment. We agree that including the perspectives of healthcare providers and administrators would provide a more comprehensive understanding of the palliative care context. We have conducted a separate qualitative study on the experiences of professionals and administrators before studying patients and caregivers and now considered for publication on PLOS ONE. Due to the volume and depth of data, it was not feasible to include both patient and provider perspectives in a single manuscript. |
| 6. Assumptions and references for sample size calculation are missing. | Thank you for your valuable comments. We have revised the **Methods** section to include the sample size calculation and the underlying assumptions, enhancing clarity and transparency regarding our study design. This revision also addresses a similar point raised by Reviewer #1, and we have made efforts to present it more clearly (**Line number** **143-149 of the manuscript)**. |
| 7. For assessing the factors associated with patient satisfaction, it needs to have proper exploration /defining of confounders, exposure, and mediators. | Thank you for this important comment. We have carefully considered potential confounders, exposures, and mediators in our analysis. The linear regression model was conducted after checking all relevant assumptions, including multicollinearity, normality, and homoscedasticity. Variables included in the model were selected based on both theoretical relevance and prior evidence, allowing appropriate adjustment for confounding and proper estimation of associations with patient satisfaction. For example, in the simple linear regression analysis, employment status and diagnosis type were significantly associated with satisfaction, their effects were no longer significant after adjusting for other covariates in the multivariable model. |
| Reviewer #3 |  |
| 1. It will be useful to describe any existing policy on Palliative care in Ethiopia and opioid availability status | Thank you for your valuable feedback and the opportunity to revise our manuscript. Now, we have included the following in the introduction section “Ethiopia has formally recognized palliative care within national health strategies and has endorsed palliative care guideline, a five years strategic plan 2025-2029 and included pain as the 5^th^ vital sign. Despite oral morphine being included in the national Essential Medicines List, access to opioid analgesics remains extremely limited, and consumption is among the lowest globally, at <3mg/person/year, compared with a global average of approximately 32mg/person per year. This persistent disparity reflects regulatory, supply chain, and health system constraints and contributes to substantial under-treatment of pain among palliative care populations.”(Line number 87-93). |
| 2. a description about the multidisciplinary team providing palliative care and type of services and any home care provided to know the background | Thank you once again for valuable comment and now added few sentences regarding MDT palliative care and home-based palliative care in the introduction section (refer line number: 93-98). |
| Reviewer #4 |  |
| 1. Abstract – Mention the tool used in the study | Thank you for your valuable feedback and the opportunity to revise our manuscript. Now we have mentioned the tool in the abstract section. |
| 2. Justify why explanatory mixed methods was chosen and not exploratory model. | Thank you so much for the insightful comment. Yes, an explanatory mixed methods design was chosen because quantitative findings formed the primary focus of the study, and qualitative data were collected subsequently to explain and contextualize these results. The research questions aimed first to quantify patterns, associations, and gaps in palliative care and opioid use, and subsequently to explore patients/caregivers perspectives to clarify observed quantitative findings. In contrast, an exploratory mixed methods design was not appropriate, as the study did not seek to generate new constructs or instruments but rather to explain and deepen understanding of predefined quantitative outcomes. |
| 3. How the sample size of 848 was arrived at? Was the sample decided based on any previous literature? | Thank you for this important observation. We would like to clarify that the reported sample size of 848 was a clerical error; the correct final sample size was 844 participants. The sample size was calculated using a single population proportion formula, informed by previous literature indicating that, in the absence of reliable national estimates, a conservative prevalence of 50% is appropriate to maximize sample size, with a 95% confidence level and a 5% margin of error. An additional allowance for non-response was included. Moreover, to maintain the desired statistical power and confidence level in the multistage sampling design (selection at department and subsequently at unit level), a design effect of 2 was applied, resulting in a final sample size of 844. Now, we have included a brief summary in the method section (**Line number** **143-149 of the manuscript).** |
| 4. 844 patients had been selected for the study – what inclusion / exclusion criteria were followed? | Thank you for the comment. As outlined in the Methods section, the inclusion criteria were: adults aged ≥18 years with a confirmed cancer diagnosis, who were able to communicate effectively, willing to participate, and available during the study period. Participants not meeting these criteria were excluded from the study. |
| 5. When SATMEDQ is not specific to assess satisfaction of palliative care services, why was the considered for the study? | Thank you for your thoughtful comment. Although the SATMED-Q is not specifically designed for palliative care patients, it comprehensively addresses both system- and treatment-level aspects of patient satisfaction, which are relevant to our study objectives. The research team conducted a face validation of the tool with this patient population and recommended its use. However, since the tool was not specifically developed for this group of patients, we have acknowledged this as a limitation in the manuscript, last paragraph of the discussion section (**line number 493-497)** |
| 6. Tool – Duration of administration? How many interviewers administered the tool? How was inter observation variation sorted out? | Thank you so much for the important question. The questionnaire was administered in approximately 7-minutes on average that ranged from 5-12 minutes. Five trained MSc-level health professionals conducted the interviews. To ensure consistency and minimize inter-observer variation, all interviewers underwent standardized training on tool administration, and completed questionnaires were regularly supervised and cross-checked throughout the study. |
| 7. Who conducted the qualitative data collection? Whether same person conducted both quantitative and qualitative data collection? | Thank you for this important question. The qualitative data were collected by the first author (AMF), who also participated in the administration of the quantitative survey. AMF has received advanced training in both qualitative and quantitative research methods, and steps were taken to minimize potential bias, including following a structured interview guide, maintaining reflexive field notes, and ensuring that data coding and analysis were conducted systematically. |
| 8. SPSS version 27 – mention the developer/company name and location | Thank you for the helpful suggestion. We have now specified that, “All data analysis was performed using SPSS **version 27 (IBM Corp., Armonk, NY, USA).” (Line number 198-199)** |
| 9. Tables show data for 844 patients (Sample Size - 848) | As we indicated earlier, it was a clerical error and correct sample size is 844. |
| 10. The Mean satisfaction level was 56.7 - how it was determined to be moderate? | We would like to clarify that we did not classify the mean satisfaction level of 56.7% as “moderate” in the manuscript; it was reported descriptively without assigning a categorical interpretation. |
| 11. Kindly clarify why the global satisfaction was high despite lower satisfaction in other domains such as side effects and daily activity. | Thank you for the comment. Based on day-to-day observations and patient interactions, it appears that patients tend to be particularly concerned about side effects and the impact of treatment on daily activities, which may sometimes be difficult to distinguish from symptoms of disease progression. In contrast, when asked about overall care, the global satisfaction domain may reflect cultural tendencies in Ethiopia, where patients often express gratitude and positive perceptions of healthcare in general. We acknowledge that this interpretation is based on informal observations and may not be generalizable, but it provides contextual insight into the patterns observed in our study. |
| 12. What qualitative data findings were matching with quantitative results? | Thank you for this comment. We have now presented the triangulated findings, highlighting where qualitative data align with the quantitative results, and summarized these in **Table 6 and line number 399-407.** |
| 13. How caregiver satisfaction (qualitative) was matched with patient satisfaction (quantitative)? | Thank you for this important question. Caregiver perspectives on satisfaction were explored qualitatively and triangulated with patient-reported quantitative satisfaction scores, identifying convergence in areas such as communication, staff responsiveness, and overall perceived quality of care. We acknowledge, however, that caregiver satisfaction was not measured quantitatively, which is noted as a study limitation **(Line number 503-506).** |
| 14. Which confounders were adjusted in regression model? | Thank you for this comment. In the simple linear regression analysis, employment status and diagnosis type were significantly associated with satisfaction, their effects were no longer significant after adjusting for other covariates in the multivariable model. |
| 15. Why only 9 out of 149 patients with severe pain were getting morphine? What were the reasons for this less number? | As we clearly indicated in the discussion section and as participants noted the qualitative part, access to morphine is a major problem, with no private or government supplier providing the product during our study period and for nearly a year prior. |
| 16. Table 1 = only 217 patients needed palliative care. Then in Table 2 = 408 were found to be on palliative care drug treatment - why the tool was administered to rest of the patients? | Thank you for this important observation. In Table 1, the term “palliative” is used to indicate the intent of treatment in cancer care. However, palliative care, as defined by the WHO, encompasses much more than treatment intent, including early integration of symptom management, psychosocial and spiritual support, and communication such as breaking bad news. All patients diagnosed with cancer require some level of palliative care, ranging from basic supportive interventions to comprehensive, multidisciplinary management. |
| 17. Key Informant Interviews   Financial constraints for Radiation / Chemotherapy are mentioned – Does it mean the participants were cancer patients needing curative care and not palliative care? Similarly, CT / MRI are mentioned – justify why they were considered for palliative care | Thank you for this important question. As noted earlier, radiation and chemotherapy may be indicated for both curative and palliative purposes. Palliative care is relevant to all cancer patients, regardless of whether the treatment intent is curative or palliative, as it addresses symptom management, psychosocial support, and overall quality of life throughout the disease trajectory. |
| 18. Reference   To follow Vancouver style guidelines – 6,7,9,10,12,18,19,21,27,28,31,32,33 | Thank you for the suggestion. We have revised the references to ensure full compliance with Vancouver style for all cited sources, including references 6, 7, 9, 10, 12, 18, 19, 21, 27, 28, 31, 32, and 33 |
| General Queries   19. Were the services at TASH chargeable? | Yes, services at TASH were chargeable; however, the source of payment varied, including out-of-pocket payments or health insurance coverage. |
| 20. What was the understanding of participants about palliative care? | As described in the qualitative findings, participants’ understanding and experience of palliative care at TASH was modest, reflecting a combination of appreciation for the services provided and concerns regarding unmet needs. |
