# Supplementary material for: Experience and satisfaction towards palliative care in an Ethiopian tertiary care setting: A mixed methods study of patients with cancer and caregivers
Source: PLOS Glob Public Health. 2026 Apr 21;6(4):e0005754. doi: 10.1371/journal.pgph.0005754 (PMC13098945; doi:10.1371/journal.pgph.0005754)
Supplement: Attachment — Submitted filename: Point by point response April 03, 2026.docx [file pgph.0005754.s006.docx]

**April 3, 2026**

**To: PLOS Global Public Health**

**Academic editors**

**Re: Response to Editor; Manuscript ID PGPH-D-25-02751R1**

**Dear Editor,**

Thank you for your careful review of our manuscript entitled *“Experience and satisfaction towards palliative care in an Ethiopian tertiary care setting: A mixed methods study of patients with cancer and caregivers”* (Manuscript ID: PGPH-D-25-02751R1). We are grateful for the constructive feedback and the opportunity to revise our work.

We have carefully addressed all the editor’s comments and revised the manuscript accordingly. All changes have been incorporated and highlighted in the tracked version of the manuscript. A detailed, point-by-point response is provided below.

**Comment 1:** *The study is only on cancer patients. Therefore, the justification should be appropriately modified. The objectives and the conclusion should also be stated specifically mentioning the study population as cancer patients.*

**Response:** Thank you for this important observation. We agree that the manuscript should consistently and explicitly reflect the study population.

- **Introduction:** We have revised the justification to clearly specify that the study focuses on palliative care among **patients with cancer** in Ethiopia.
- **Objectives:** The study objectives have been refined to explicitly state that the research pertains to **patients with cancer and their caregivers**.
- **Conclusion:** The conclusion has been revised to clearly reflect that the findings are specific to **patients with cancer**, ensuring alignment with the study population.

These revisions have been made throughout the manuscript to improve clarity and consistency.

**Comment 2:** *Language correction is needed throughout the manuscript.*

**Response:**
We appreciate this recommendation and have thoroughly revised the manuscript to improve language clarity, grammar, and overall readability. Specifically:

- The manuscript has undergone comprehensive **professional language editing**.
- Grammatical errors, sentence structure, and phrasing have been improved across all sections.
- Redundant wording has been minimized, figures and tables are as per PLOS guide and clarity has been enhanced in line with **PLOS Global Public Health** style guidelines.

We believe that these revisions have significantly strengthened the manuscript and addressed all remaining concerns. We sincerely appreciate your time and consideration and hope that the revised version meets the journal’s requirements for publication.

Thank you for your consideration.

**Kind regards,**

**Atalay M Fentie (AMF)**

(On behalf of all authors)
