# Supplementary material for: Experience and satisfaction towards palliative care in an Ethiopian tertiary care setting: A mixed methods study of patients with cancer and caregivers
Source: PLOS Glob Public Health. 2026 Apr 21;6(4):e0005754. doi: 10.1371/journal.pgph.0005754 (PMC13098945; doi:10.1371/journal.pgph.0005754)
Supplement: Attachment — Submitted filename: PLOS Reviewer comments.docx [file pgph.0005754.s003.docx]

**Experience and satisfaction towards palliative care in an Ethiopian tertiary care setting: A mixed methods study of patients with cancer and caregivers**

Dear Authors

- Interesting Study
- Statistical methods applied add strength to the findings of the study

Kindly address the following queries and suggestions

- Abstract – Mention the tool used in the study
- Methodology
- Justify why explanatory mixed methods was chosen and not exploratory model
- How the sample size of 848 was arrived at? Was the sample decided based on any previous literature?
- 848 patients had been selected for the study – what inclusion / exclusion criteria were followed?
- When SATMEDQ is not specific to assess satisfaction of palliative care services, why was the considered for the study?
- Tool – Duration of administration?
- How many interviewers administered the tool?
- How was inter observation variation sorted out?
- Who conducted the qualitative data collection? Whether same person conducted both quantitative and qualitative data collection?
- SPSS version 27 – mention the developer/company name and location
- Results
- Tables show data for 844 patients (Sample Size - 848)
- The Mean satisfaction level was 56.7 - how it was determined to be moderate?
- Kindly clarify why the global satisfaction was high despite lower satisfaction in other domains such as side effects and daily activity
- What qualitative data findings were matching with quantitative results?
- How caregiver satisfaction (qualitative) was matched with patient satisfaction ( quantitative ) ?
- Which confounders were adjusted in regression model ?
- Why only 9 out of 149 patients with severe pain were getting morphine? What were the reasons for this less number?
- Table 1 = only 217 patients needed palliative care. Then in Table 2 = 408 were found to be on palliative care drug treatment - why the tool was administered to rest of the patients?
- Key Informant Interviews

Financial constraints for Radiation / Chemotherapy are mentioned – Does it mean the participants were cancer patients needing curative care and not palliative care?

- Similarly, CT / MRI are mentioned – justify why they were considered for palliative care
- Reference

To follow Vancouver style guidelines – 6,7,9,10,12,18,19,21,27,28,31,32,33

- General Queries
- Were the services at TASH chargeable?
- What was the understanding of participants about palliative care?

==========================================================
